# Material Origins of the Accelerated Operational Wear of RD-33 Engine Blades

**DOI:** 10.3390/ma14020336

**Published:** 2021-01-11

**Authors:** Adam Kozakiewicz, Stanisław Jóźwiak, Przemysław Jóźwiak, Stanisław Kachel

**Affiliations:** 1 Faculty of Mechatronics, Armament and Aerospace, Institute of Aviation Technology, Military University of Technology, 00-908 Warszawa, Poland; adam.kozakiewicz@wat.edu.pl; 2 Faculty of Advanced Technology and Chemistry, Institute of Materials Science and Engineering, Military University of Technology, 00-908 Warszawa, Poland; stanislaw.jozwiak@wat.edu.pl; 3 Air Force Institute of Technology, 01-494 Warszawa, Poland; przemyslaw.jozwiak@itwl.pl

**Keywords:** turbine jet engine, material tests, ember-resistant alloys

## Abstract

The structural and strength analysis of the materials used to construct an important engine element such as the turbine is of great significance, at both the design stage and during tests and training relating to emergency situations. This paper presents the results of a study on the chemical composition, morphology, and phased structure of the metallic construction material used to produce the blades of the high- and low-pressure turbines of the RD-33 jet engine, which is the propulsion unit of the MiG-29 aircraft. On the basis of an analysis of the chemical composition and phased structure, the data obtained from tests of the blade material allowed the grade of the alloy used to construct the tested elements of the jet engine turbine to be determined. The structural stability of the material was found to be lower in comparison with the engine operating conditions, which was shown by a clear decrease in the resistance properties of the blade material. The results obtained may be used as a basis for analyzing the life span of an object or a selection of material replacements, which may enable the production of the analyzed engine element.

## 1. Introduction

**Figure 1:**
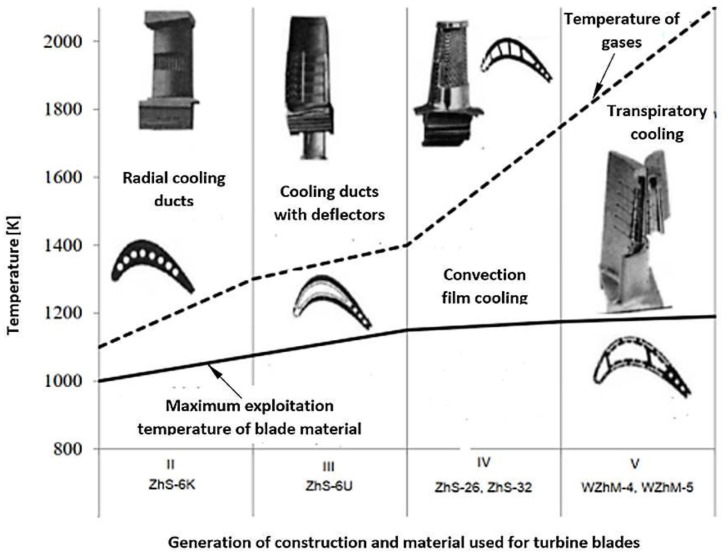
Development of the cooling technology and change in materials used to construct Russian turbine jet engines [3].

Aviation turbine engines must meet very high requirements relating to reliability, strength, minimum weight, serviceability, the acceptable period of use in service, noise, ecology, and cost-effectiveness [1]. In turbine jet engines, one of the essential components that must meet these criteria is the turbine assembly. A turbine assembly is a system that is heavily loaded mechanically and thermally. This is caused by tension and bending of the blades as a result of centrifugal forces, bending, and twisting resulting from the exhaust gas mass flow. It is also caused by high temperature. This results in a number of complex stress states, especially in the blade palisades of rotor wreaths, including the occurrence of variable stress. Therefore, in order to increase the operational properties, two changes in the blade design are made: changes in the design that improve the cooling efficiency and changes in the material of the blade matrix and protective coatings that protect from overheating (Figure 1). One particular question that arises when it comes to the employment of aircraft jet engines is what should be done to prolong the engine’s service life, specifically the lifespan of the engine’s turbine. The problem becomes more critical when the engines in question are used and replaced on the basis of hours in service. Complicating this problem are factors concerning the engine component quality and fatigue. A good example of this would be the Klimov RD-33 engine, used by the Polish Air Force. If we analyze the operating costs of two RD-33 engines that power the MiG-29 in comparison with those of the F-100-PW-229 engine used to power the F-16C, the costs of repairs are much higher in the case of the RD-33 (about 18 million dollars in total costs [2]), considering an operational lifespan of approximately 3200 h for both units.

These costs are further increased in the event of premature engine failure, resulting in removal, as occurred in the case we analyzed. For instance, approximately 13% of engines are prematurely decommissioned due to damage to the high-pressure turbine [4]. Studies have found that the most common causes of turbine element damage are mechanical and high-temperature damage [5]. The main mechanics behind mechanical damage are the propagation of cracks, specifically fatigue-related ones, in addition to abrasive wear on the surface of turbine blades [6,7]. However, based on S. Szczeciński’s papers [8], the problem of abrasive wear applies mostly to turbine engines installed in helicopters, which often use unimproved “dusty” airfields. This paper focuses on turbofan engines fitted to attack aircraft, which generally operate at improved airfields, where the problem of dust and foreign object damage is notably less of a concern. Moreover, S. Szczeciński claims that low-bypass turbofan 2-spool jet engines—the analyzed RD-33 being an example of such an engine due to the placement of the turbine within the profile of the core of the engine—are less susceptible to abrasive wear of the turbine’s elements [8]. The development of cracks on the leading edge or the trailing edge of the turbine blade is usually caused by changes in the structure of the blade material both in diffusion zones of the coating of the core and in the overheated core (HAZ), which lead to the creation of hard and fragile phases in grain boundaries, resulting in a lowered fatigue strength due to an easier generation of cracks [9]. The cause of changes in the material structure is usually damage to thermal barrier coatings (TBC), which occurs due to high-temperature gaseous corrosion [10,11]. Based on the analysis in [12], the most common cause of first-degree damage of the turbines in RD-33 engines is high-temperature gaseous corrosion (totaling 45% of all kinds of damage), while the development of cracks is the cause in only 35% of cases. In light of the abovementioned facts, this paper focuses on structural changes caused by turbine blade overheating, which might result in various damage mechanisms, including accelerated fatigue of the material. Accelerated wear of engine components generates higher costs of maintenance, and many attempts at elucidating the causes of damage and finding preventive measures have been made, although it has been impossible to acquire precise data from the manufacturer. Additionally, the turbine blades of the RD-33 engine are made of various types of high-temperature superalloys, with a variety of thermal performance characteristics. It was therefore necessary to determine the properties of the materials used in the turbine blades, after the engine’s premature removal from service, in order to accurately analyze the mechanisms underlying their damage. This analysis would imply the benefit of modifications to the thermal barrier coatings, effectively expanding the service time of the engine.

## 2. Analysis of the State of the Issue

The basic parameter characterizing the ember-resistance of nickel-based super alloys, intended for the construction of the turbine blades of jet engines, is their creep resistance. The increase of this parameter, depending on the phased structure of the alloy, and in particular, the share of γ/γ’ phases, reinforcement carbides, Laves phases, etc., is obtained by modifying the chemical composition and morphology of the crystal structure of the blade matrix material. The same also applies in the case of Russian superalloys. An increase in the creep resistance of the materials used to produce turbine blades was obtained by using Re and Ta as additives, which, together with directional crystallization (ZhS-26) or monocrystalline crystallization (ZhS-32), allowed, unlike ZhS-6U alloy, for a significant increase of this parameter (Table 1).

An increase of the creep resistance of ZhS-32 alloy, according to the authors in [13], allowed the temperature of the gases on the blades in the first stage of the turbine to be increased from 1263 °C (for the ZhS-6U-VI alloy [14]) up to 1400 °C. However, due to the acceptable period of use in service of the RD-33 engine, the prolonged temperature of the blade material, exceeding 1100 °C, is not recommended.

**Figure 2:**
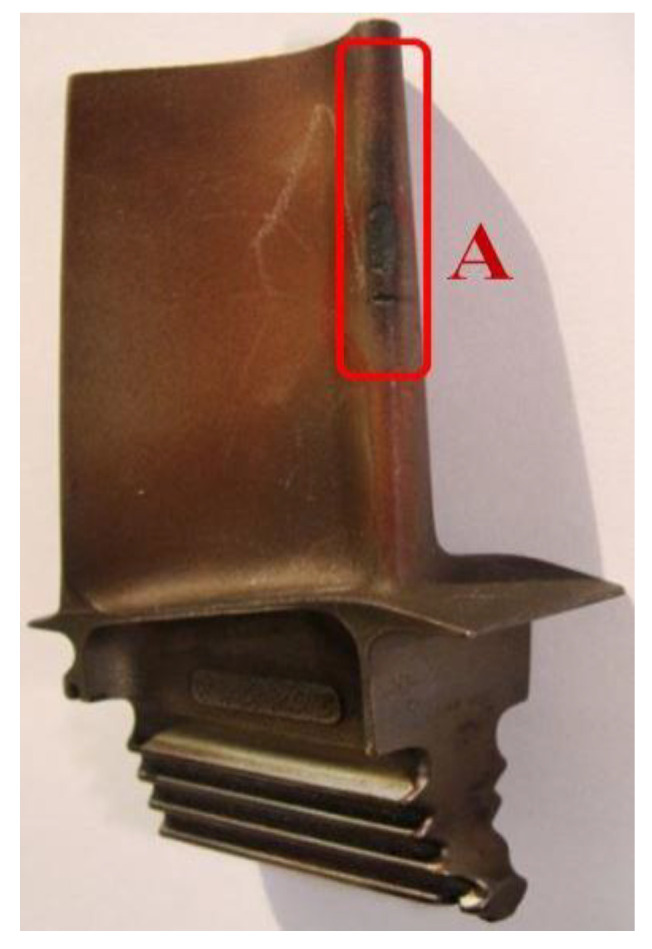
Blade from the rotor wreath of a high-pressure turbine, with a marked zone (A) of gas corrosion damage and crack damage of the leading edge of the blade.

The preservation of these exploitation regimes allowed the acceptable period of use in service of the RD-33 engine blades made of ZhS-32 superalloy to be extended from 300 h (ZhS-6) up to 1000 h of exploitation. Nevertheless, one must keep in mind that prolonged exposure to hot exhaust gases can lead to structural changes within the blade material, especially if the protective coating is damaged, thus leading to its destruction. A detailed analysis of the RD-33 engine damage showed that over 42% of failures were caused by damage to the blades, and damage to blades in the first stage of the turbine accounted for over 60% [13]. The main causes of damage are gaseous corrosion at the leading edge, cracks at the leading and trailing edges, and thermo-mechanical damage (Figure 2).

**Figure 3:**
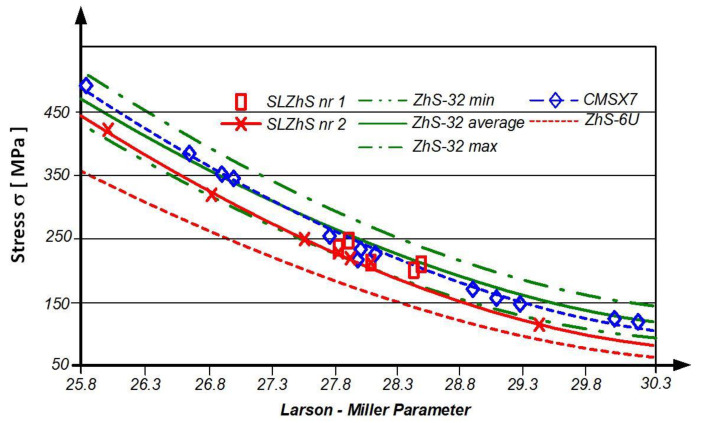
Influence of temperature and exploitation time on the exploitation stress of ZhS alloys used in the construction of RD-33 engine turbine blades, as defined by the Larson–Miller parameter P [3].

The scope of application of the alloys used to construct the blades of the RD-33 engine and, in particular, the creep resistance, depending on the temperature and duration of its long-term impact on the material, are best represented by the Larson–Miller parametric coordinates, as defined by Formula (1) (Figure 3):

P = (T + 273) × (20 + log t) ∗ 10^−3^ (1)

where:

T—temperature [°C]
t—exploitation time [h]

**Figure 4:**
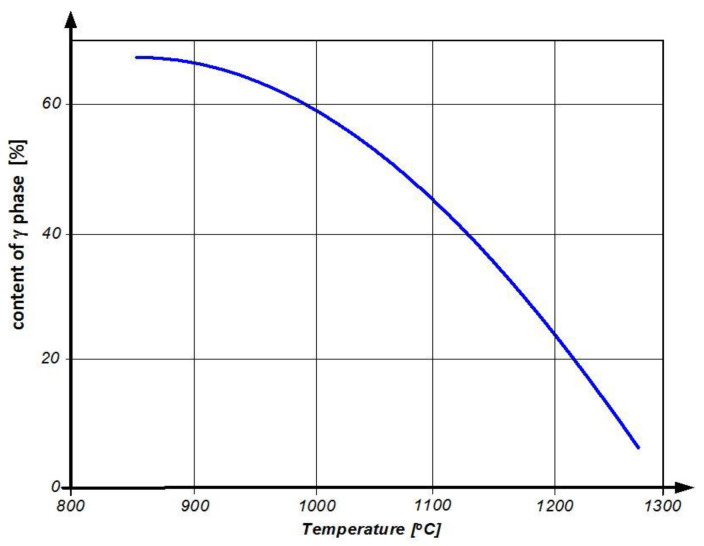
Influence of exploitation temperature on the share of phase γ’ in the structure of the ZhS-32 alloy [14].

The higher permissible exploitation stress of the ZhS-32 alloy, compared to ZhS-6U, should be explained by its higher volume content of phase γ’ in the material structure, which is 67% (compared to 57%), and its higher thermal stability, caused by the Re and Ta additives, which increase the temperature of the solidus from 1240 °C (ZhS-6U) up to 1310 °C (ZhS-32) [15]. It is obvious that the long-drawn process of exploitation, linked with the high-temperature influence of exhaust gases, causes the grain coarsening and decrease of the phase γ’ content in the blade material structure (Figure 4).

**Figure 5:**
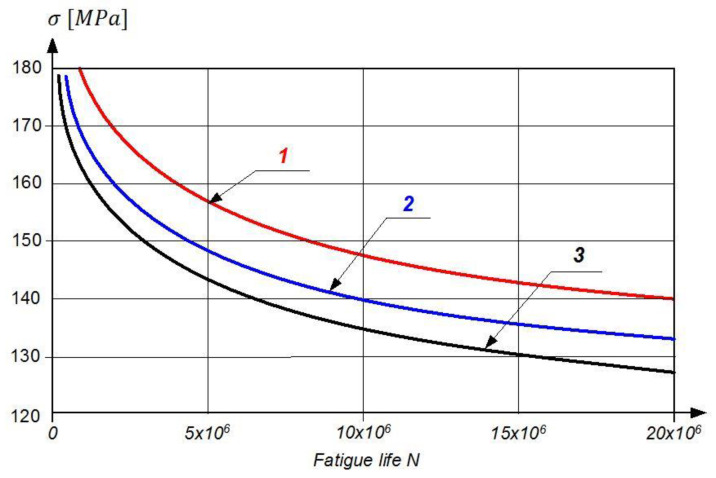
The fatigue curves of turbine blades made of EI-437B, where the working time t = 0 h (1), t = 200 h (2), and t = 400 h (3) [15].

These phenomena result in a decrease in strength parameters in nickel-based super-alloys, manifested by a significant decrease in the fatigue strength of the blade material (Figure 5).

In connection with the abovementioned conditionalities/exploitation and strength rationale, for the purpose of research on the construction of the aircraft engine assemblies in the area of structural analysis, a thorough analysis of material degradation shall be carried out. This must take into account tests of the grain structure and the chemical composition that determines the phased structure, which directly affects the operational properties, including the mechanical properties that determine the performance of such a complex structure as the modern turbine system. Incorrect use of a given construction material, or incorrect exploitation or other factors causing the limits (resulting from strength calculations) of the applied materials to be exceeded, may lead to a reduction in strength. This, in turn, may lead to damage to the turbine assembly and engine.

The above assumptions form the basis for the research undertaken to determine the causes of premature damage to the blades of the turbines of the RD-33 engine of the MiG-29 airplane, resulting in the necessity to dismount the engines and perform engine overhauls.

## 3. Sample Preparation and Test Methodology

**Figure 6:**
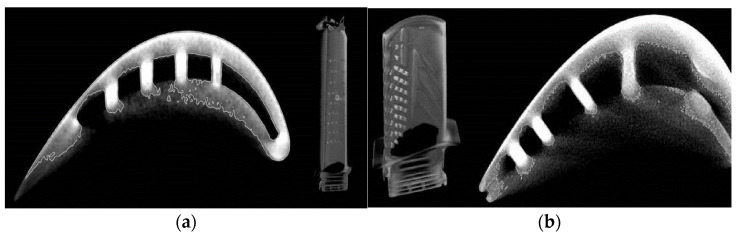
The shape of the LPT blade (**a**) and the HPT blade (**b**), with visible cooling channels, obtained by the computer-based micro-tomography CT.

The mass loads and, in particular, the heat loads of the turbine blades caused by the exhaust gas stream from the combustion chamber (1560 K) require cooling of this engine component. In the RD-33 motor, under analysis, the blades of both the LPT and HPT are cooled by means of internal channels (Figure 6), and the blades of the HPT, due to the higher gas temperature, also have channels for cooling the trailing edge.

**Figure 7:**
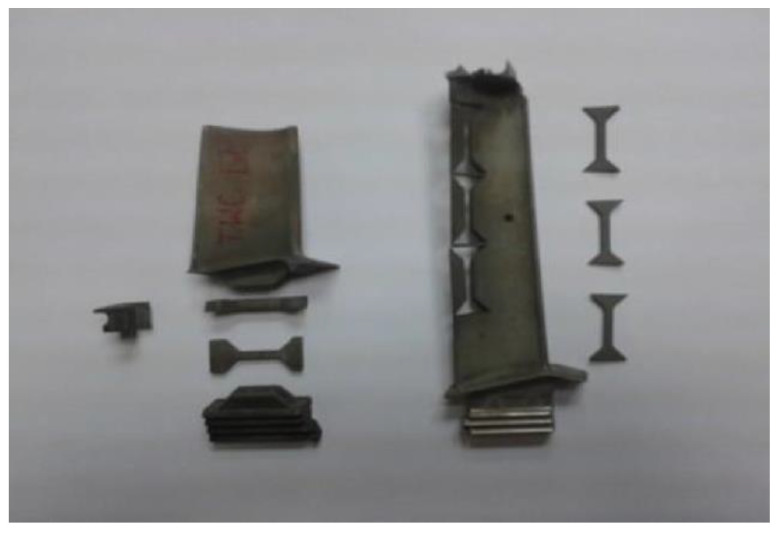
View of specimens cut out (using the electro-discharge method) of a material of the LPT blade and view of specimens for static tensile testing cut out of the material of the HPT blade and LPT blade.

This blade design poses serious problems relating to the selection of a place to take the strength test specimens in order to properly perform a static tensile test. Therefore, taking into account the nature of the mechanical loads carried by the material of the component tested, it was assumed that the specimens for the static tensile tests will be cut along the blade feather of the LPT, i.e., in the direction of the centrifugal forces and, in the case of the HPT, from the lock material, i.e., the component subjected to the highest mass loads (Figure 7).

Nevertheless, the three-dimensional shape, with variable cross-sections and curvatures, made it impossible to clamp and thus to make the strength specimens with classical machines for cavity machining (edging method machining). Therefore, in order to preserve the assumed shape, dimensions, and geometric quality of the surface of the specimens, as well as to minimize the impact of the preparation process on the structural changes of the blade material, electro-discharge machining was used as the cutting method. All of the samples were made using the electro-discharge wire rod numerically controlled machine, and the assumed geometric dimensions have been respected.

**Figure 8:**
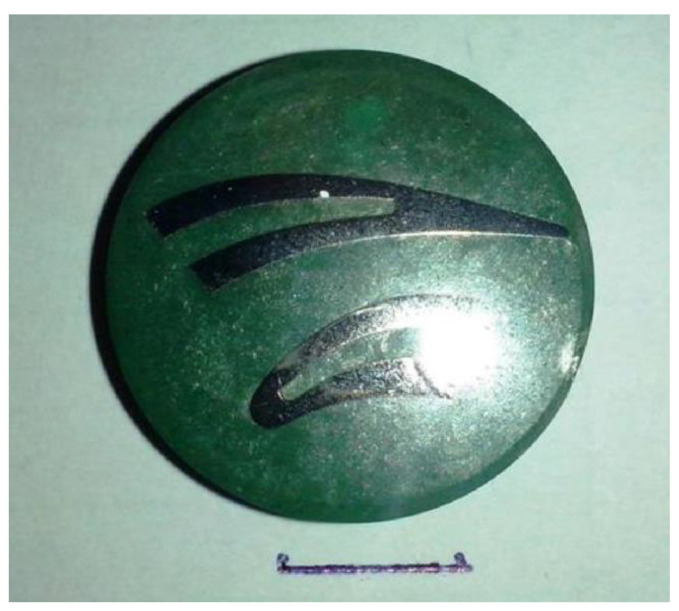
Metallographic specimen of the material of the LPT blade.

In order to make microscopic observations to reveal the grain structure of the blade material, metallographic samples were prepared (Figure 8). They were prepared, first, by mounting them in a conductive resin and then grinding them with abrasive papers of varying granulations in the range of 100–1000 and, second, by a final polishing with a diamond slurry of polishing powder using the Struers polishing machine.

Microscopic observations at magnifications of 100–10,000 times were made by using the Quanta 3 D FEG (SEM/FIB) high-resolution scanning microscope, which, in addition to grain structure morphology analysis, also enables, with the use of EDS/WDS/EBSD attachments, complex studies of the chemical composition in micro-areas and the analysis of crystallographic orientation.

Since chemical composition microanalysis indirectly allows only for the identification of the phased structure of the material under investigation, it was necessary to perform X-ray phase analysis (XRD). Identifying the type of crystal lattice and measuring the parameters of the elementary crystal cell allows, in a precise and unambiguous way, for the identification of the phased structure of the material under investigation, on which the functional properties, including the strength properties, depend. The phase X-ray analysis was carried out with the ULTIMA IV Rigaku diffractometer using a parallel beam. The measurements were carried out using a cobalt lamp, with a radiation length CoKα of 1 = 1.78892 Å, power of 1600 W, scanning step of 0.02°, and scanning speed of 2°/min in the angular range of 2 Θ = 20–140°. The identification of the obtained reflections and phased analysis were carried out on the basis of the crystallographic database, PDF-4.

The evaluation of the mechanical properties of the material of the tested blades was based on the hardness, microhardness, and the static tensile tests. Due to the ultrafine grain structure of the tested material, in order to unequivocally confirm the correctness of the hardness measurements, the testing of this strength parameter was carried out using three measurement methods (HBW2.5/187.5, HV10—Wolpert Wilson Testor, and HRC—Rockwell PW 106 hardness meter), and then (using appropriate nomograms, enabling a comparison of the hardness values measured by different methods) the results obtained were evaluated, taking into account the statistical analysis to determine the correctness of the data obtained. In order to further assess the homogeneity of the matrix structure, Vickers microhardness tests were performed in various areas of the blades using the SHIMADZU-DORERNST M hardness tester, with a load of 25 G.

**Figure 9:**
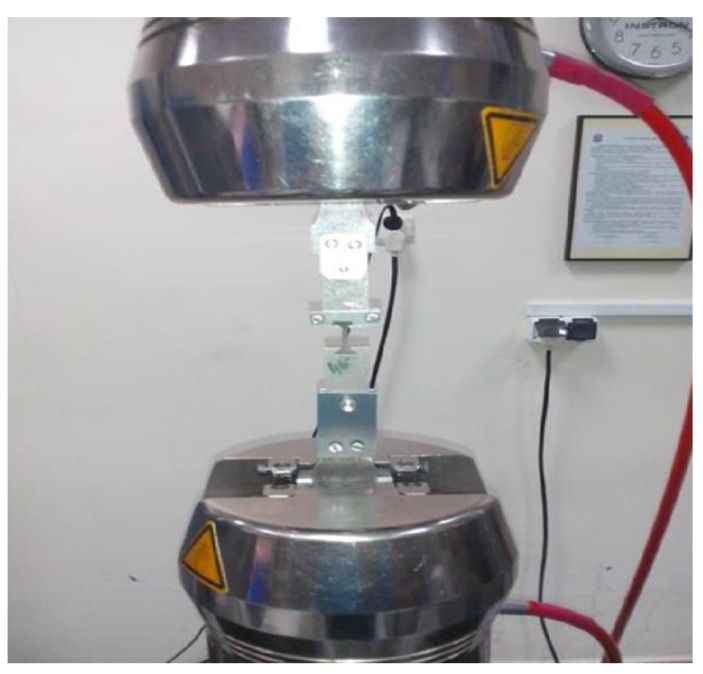
View of the test sample in specially designed holders.

A static tensile test, considered a destructive test, was carried out with the Instron 8501 Plus universal testing machine in accordance with the PN-EN ISO 6892–1:2010 standard. Due to the non-standardized, small dimensions of the strength specimens obtained (Figure 7), in order to execute the static tensile test, it was necessary to design and manufacture special holders to give the test a proper performance (Figure 9).

The manufactured tooling made it possible to determine the following basic strength parameters based on the obtained tensile curves:

- 0.2% offset yield strength R_0,2_;
- ultimate tensile strength R_m_;
- fracture strain of sample A;
- Young’s modulus E.

## 4. The Results of the Tests

**Figure 10:**
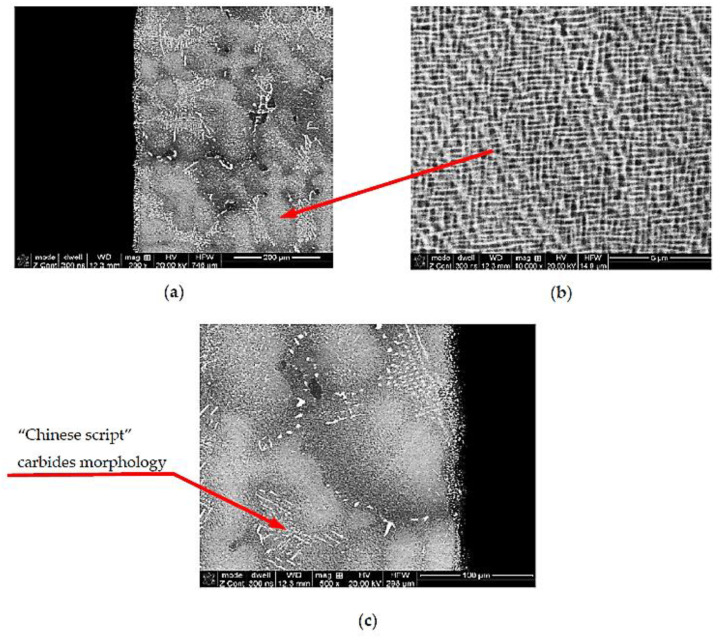
Multiphase structure of the TWC blade material built on the matrix of the γ phase (**a**), with cuboidal precipitates of the intermetallic phase γ’ (**b**) and reinforcing carbide precipitates (**c**).

The microscopic observations carried out at magnifications of 100–10,000× (Figure 10) showed that the TWC blade material is characterized by a multiphase structure typical for nickel-based heat-resistant alloys. After the supersaturation and aging process, it consists of the primary, dendritic structure, resulting from the crystallization of the alloy (Figure 10a); the solid solution γ, formed during supersaturation; cuboid intermetallic precipitates of the γ’ phase, released during the aging process (Figure 10b); and reinforcing carbides, with a “Chinese script” morphology (Figure 10c).

The available data show that the blades of the turbine of the RD-33 engine are currently made of two types of alloys: monocrystalline ZhS-32 or directionally crystallized Zhs-26.

**Figure 11:**
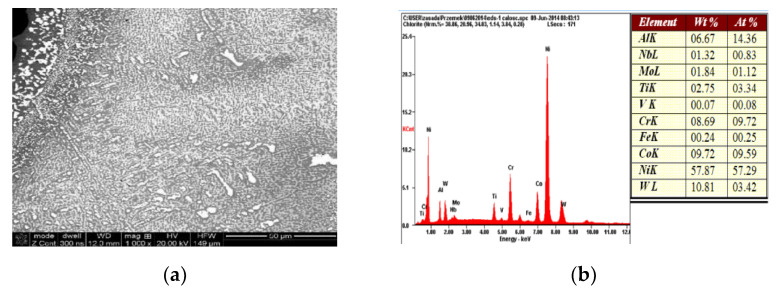
Result of the microanalysis of the chemical composition of the material of the HPT blade. The area subject to microanalysis (**a**), the spectrum of identified elements (**b**).

Nevertheless, the microanalysis of the chemical composition of the multi-phase blade structure allowed for the identification of significant discrepancies, mainly in terms of the content of chromium and titanium, between the tested material and the ZhS-32 and ZhS-26 alloys. The chemical composition of the analyzed material of the blades suggests that they are made of an alloy with a chemical composition corresponding to the heat-resistant nickel-based superalloy, MAR-M 200 [16], which also corresponds to the chemical composition of the Russian ZhS-6U alloy [17] (Figure 11) (Table 2.).

**Figure 12:**
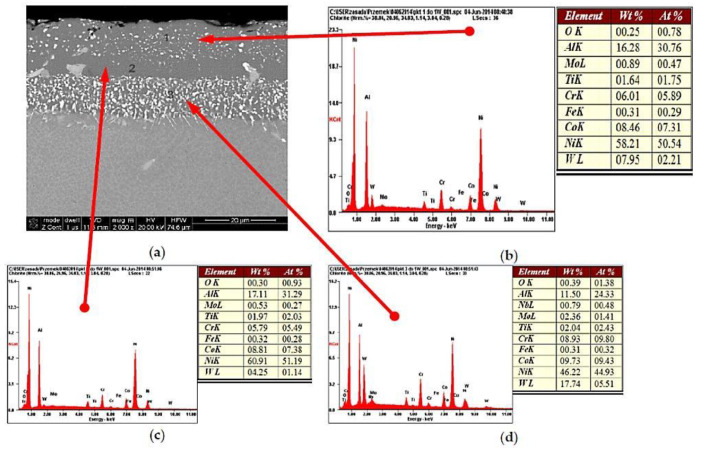
Three-layer barrier nickel aluminides (NiAl), with the coating diffusively applied to the TWC blade material (**a**), and the results of the microanalysis of the chemical composition in individual areas (**b**–**d**).

The maximum exploitation temperature of the MAR-M 200 alloy is (according to the CES Edu Pack database) in the range of 815–983 °C, and the temperature of the exhaust gases before the rotor wreath of a high pressure turbine (HPT) of the RD-33 engine reaches the upper level of the temperature range of the material used for the blades of the HPT. Therefore, in order to protect the material of the blade core, it was covered with a barrier coating, based on the NiAl intermetallic phase (Figure 12), created using diffusional aluminizing technology.

This process has led to the constitution on the surface of the blade material of a three-layer heat-resistant coating of NiAl intermetallic phase protecting the blade material from overheating.

**Figure 13:**
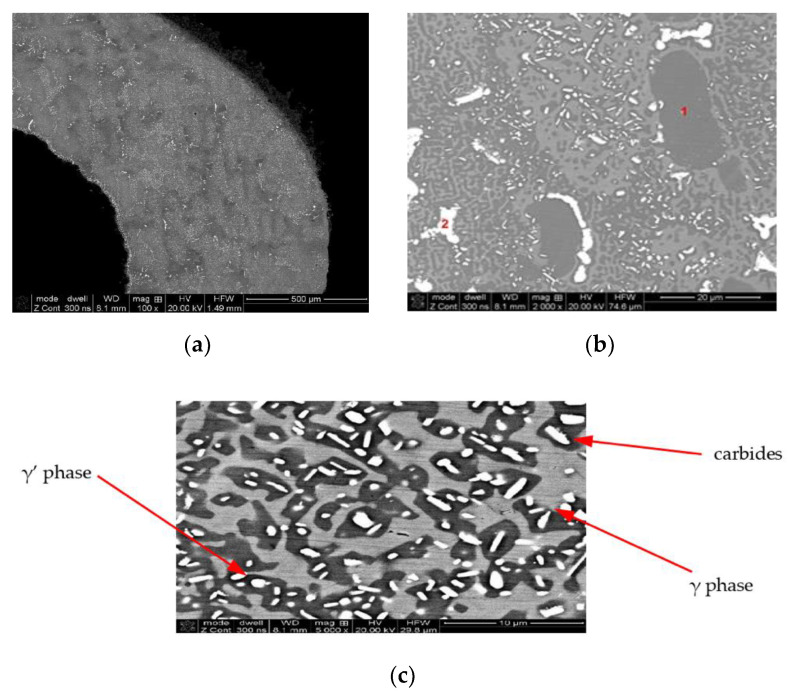
Damaged barrier coating at the leading edge of the HPT blade (**a**) and the effect of the anomalous growth of the γ’ phase (**b**) and increase in the share of carbides in the matrix of the core material (**c**) due to overheating.

Nevertheless, in spite of the material of the core of the HPT blade being protected against the temperature influence of exhaust gases, the barrier coating was damaged at the leading edge, which, due to temperature increase, led to changes in the grain structure of the blade at the overheating point (Figure 13a). In addition to a small but noticeable growth of the grains of the γ matrix from 80 up to 95 μm, an anomalous selective growth and coagulation of the precipitations of the γ’ phase (area 1 Figure 13b) growth and coagulation (area 2 Figure 13b) and an increase in the content of the carbides in the structure are also observed (Figure 13c), which may lead to a decrease of the creep resistance of the blade material.

While the less thermally loaded material of the LPT blade, coated with a diffusion barrier layer made of aluminides, is also constructed of a multi-phased structure (γ + γ’ + MC), no abnormal growth of the superstructure γ’ and carbides was observed in material of the LPT blade.

**Figure 14:**
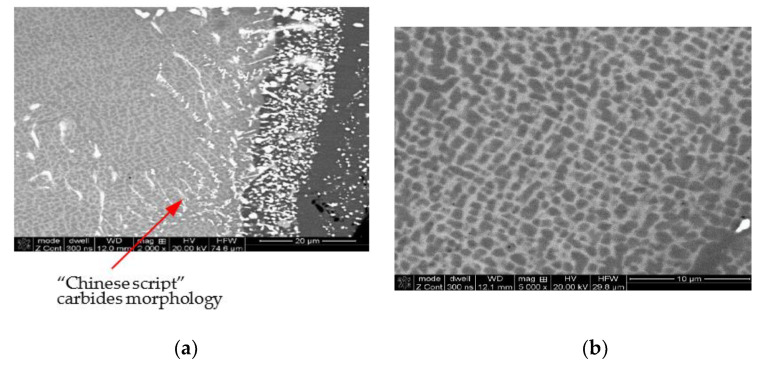

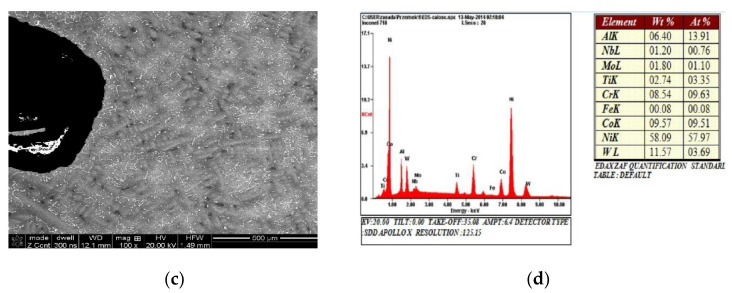
View of the structure of the LPT blade with the barrier coating (**a**), characteristic grain of the cuboidal phase γ’ (**b**), and uniformly distributed reinforcing precipitations of carbides (**c**). The result of the microanalysis of the chemical composition of the core material (**d**).

A microanalysis of the chemical composition suggests that a similar phased composition to that of the material of the HPT blade is the result of a similar chemical composition, suggesting that the same alloy was used for the material of the LPT blade, as in the case of the HPT blade (Figure 14) and (Table 3).

The conducted microscopic observations made it possible to measure, by means of microscopic image analysis, the volume content of individual phases in the structures of the analyzed blades and to assess the size of the sections of the dendrite arms of the primary γ solid solution and the average grain size of the other identified structural components’ −γ’ intermetallic phase and carbides. From the collected data presented in Table 4, it can be concluded that the structure of the material of the blades is stable so long as it is protected by a barrier coating.

The damage to the protective layer, as already indicated, leads to a noticeable growth of the matrix grains and a three-fold increase of the carbide volume content.

**Figure 15:**
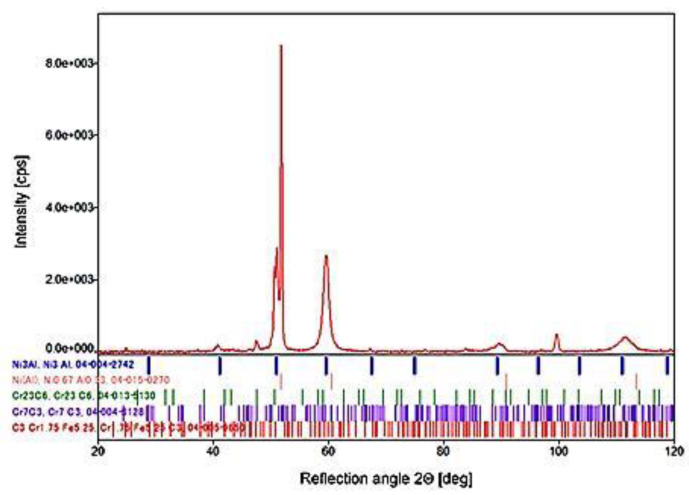
X-ray diffraction (XRD) pattern of the blade material with the identified phases.

The microscopic observations were confirmed by the XRD analysis. Based on the X-ray phase analysis measurements, by analyzing the positions of the obtained reflections, it was possible to unequivocally confirm the multi-phase structure of the blade material observed during the microscopic observations (Figure 15).

The distribution of the alloying elements suggested the occurrence of the three-phased structure of the material based on the solid solution of aluminum in nickel γ and of the areas of intermetallic phase γ’ on the matrix of the Ni_3_Al superstructure and carbide precipitates, mainly Cr_23_C_6_. This was fully confirmed by the comparative analysis of the 2 Θ angular position of the obtained diffraction reflections and the data contained in the PDF-4 database. Additional evidence confirming the comparable structural-phase composition of the analyzed blades are microhardness measurements [18] carried out on the materials of the tested blades.

**Figure 16:**
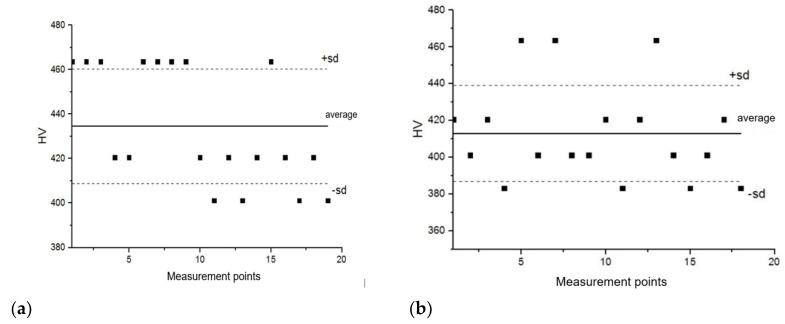

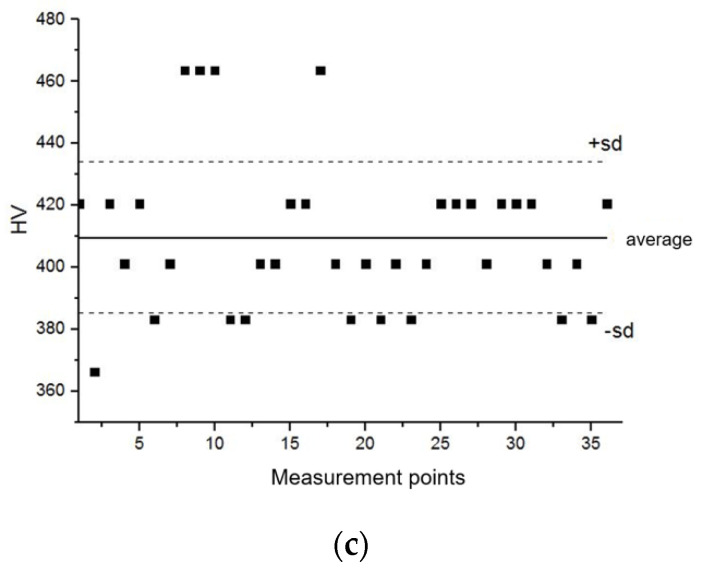
Statistical distribution of the microhardness of the material of the HPT blade in the overheated zone (**a**), in the non-overheated zone (**b**), and in the material of the LPT blade (**c**).

These tests were carried out with the semi-automatic microhardness tester, SHIMADZU-DORERNST M, and the obtained results were subjected to statistical analysis, which confirmed the structural homogeneity of the analyzed material areas, characterized by an average value of microhardness at a statistically uniform level within the range of 410–430 HV 0.025 (Figure 16).

**Figure 17:**
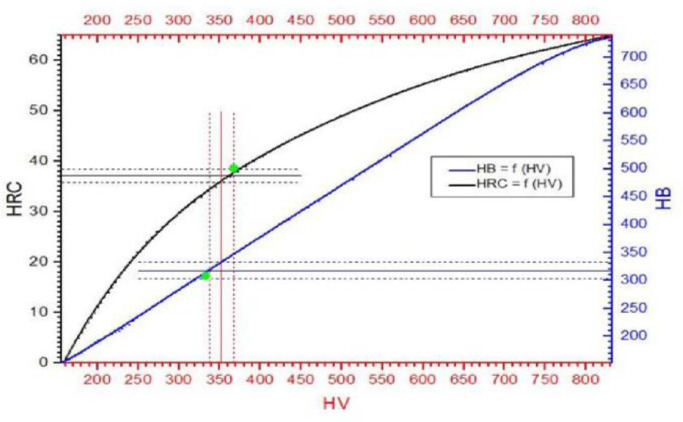
Comparison of the hardness measurements obtained with the Rockwell PW 106 hardness tester and the Wolpert Wilson Testor 751 universal hardness tester.

The observed structural changes were reflected in the strength parameters of the alloy used for the construction of the tested blades. As can be seen in Figure 17, where the hardness values obtained are marked with the determined standard deviation, the intersection points (marked in green) of the hardness obtained are located on the curves, which represent the dependency between the Brinell and Rockwell hardness as a function of the Vickers hardness (curves HB = f(HV) and HRB = f(HV)), which clearly proves the correctness of the measurements carried out. It also proves the high homogeneity of the tested material, caused by the refinement of the grain structure, despite its multiphase nature.

**Figure 18:**
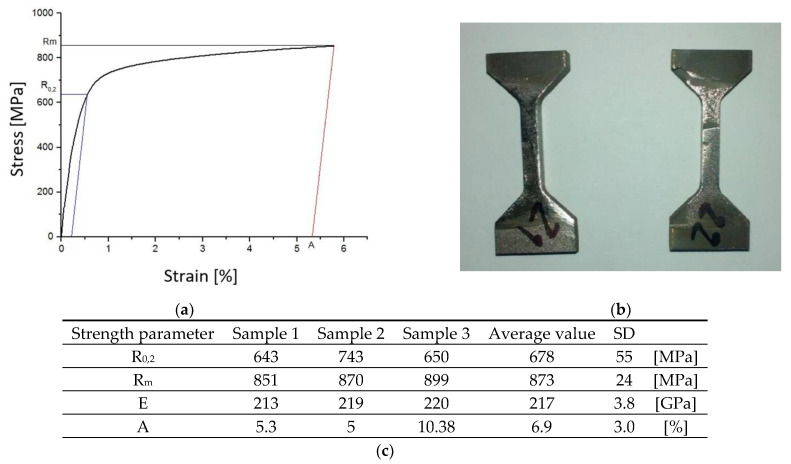
Tensile curve of the blade material (**a**), view of specimens before and after the tensile test (**b**), and the determined strength parameters (**c**).

However, the average hardness value of the analyzed material, after its exploitation, decreased to the level of 350 HV10, compared to 450 HV10, which is typical in the case of a correctly structured ZhS-6U alloy. It should also be noted that the hardness (Figure 17) and microhardness (Figure 16) values measured by the same Vickers method differ significantly, reaching 70 HV hardness units in extreme cases. However, the observed phenomenon, the indentation size effect (ISE), is in accordance with the variable hardness law, which describes the influence of elastic deformation on the value of the obtained results, i.e., the smaller the load, the greater the influence of elastic deformation and thus the greater the hardness value. At this point, it should be recalled that the load for the microhardness measurements was 25 g, and for the hardness measurements, it was 10 kg. The decrease in the strength properties is confirmed by the results of the static tensile test (Figure 18).

In accordance with the Hall–Petch relationship, the decrease in the share of the γ’ phase in the material structure and the growth there caused a decrease in the yield point from the original level of 770 MPa for the ZhS-6U alloy to 678 MPa for the material of the tested blade (Table 5).

## 5. Summary

The data presented in this study were obtained as a result of material tests of the blades of the rotors of the low- and high-pressure turbines of the RD-33 engine, taking into account the chemical composition and the phase structure corresponding to the Russian alloy, the Zhs-6U type, used in the early versions of the RD-33 engine. The deployment of blades made of the ZhS-26 and ZhS-32 alloys allowed for an increase of the temperature of exhaust gases before the turbine, thus increasing the engine performance. However, the conscious or unconscious use, under these exploitation conditions, of blades made of ZhS-6U alloy, with a noticeably lower structural stability, combined with local damage to the barrier protective coating, resulted in a distinctive reduction of the strength properties (primarily the plasticity limit) of more than 10 per cent, which could ultimately lead to engine failure and even damage to the engine.

**Figure 19:**
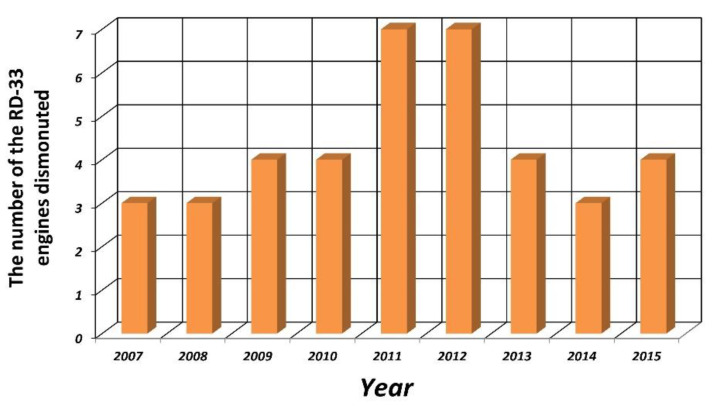
The number of RD-33 engines dismounted from airplanes due to damage to the high-pressure turbine in one of the air bases in Poland during the years 2007–2015 [4].

The values of the determined strength parameters will be used in further work related to numerical analyses in the field of strength issues concerning the life and failure of engines manufactured by Russia, including the RD-33 engine. The importance and need for such works [4,12,19] may be proved by the number of RD-33 engines dismounted from the airplanes in one of the air bases due to damage to the high-pressure turbine, which is shown in the diagram in Figure 19. In addition, the analysis of structural changes caused by exploitation conditions can be helpful in the search for and selection of material substitutes used to construct the engine element under analysis, which will meet increasingly higher, mainly temperature-related design requirements.

## Figures and Tables

**Table 1:** Creep resistance of super ZhS alloys [12].

Alloy	$\sigma_{100}^{900}\;(\mathrm{MPa})$	$\sigma_{100}^{1000}\;(\mathrm{MPa})$	$\sigma_{100}^{1100}\;(\mathrm{MPa})$
ZhS-6U	350	170	65
ZhS-26	400	200	85
ZhS-32	480	250	120

**Table 2:** Weight percentage (%) of individual alloy elements.

Elements:	Ni	Cr	Co	Fe	Al	Ti	Mo	Nb	W	Ta	Re
Tested alloy	58.49	8.68	9.86	0.77	6.19	2.70	1.75	1.26	10.69	-	-
MAR-M 200	59	9	10	<1	5	2	-	1	12.5	-	-
ZhS-6U	59.5	8.8	10.3	2	5.5	2.6	1.6	-	9.7	-	-
ZhS-32 Monocrystalline	residue	4.3–5.6	8.0–10.0	-	5.6–6.3	-	0.8–1.4	1,4–1.8	7.7–9.5	3.5–4.5	3.5–4.5
ZhS-26 directionally crystallized	residue	4.1–5.3	8.7–9.3	-	5.6–6.1	0.8–1.2	0.8–1.2	1.4–1.8	11.2–12	-	-

**Table 3:** Comparison with the microanalysis of the material of the HPT blade.

Elements	Weight Percentage (%)								
	Ni	Cr	Co	Fe	Al	Ti	Mo	Nb	W
HPT blade	58.09	8.54	9.57	0.08	6.40	2.74	1.80	1.20	11.57
LPT blade	57.87	8.69	9.72	0.24	6.67	2.75	1.84	1.32	10.81

**Table 4:** Volume content and grain size of individual phases i006E of the structure of the analyzed blades.

Phases		HPT				HPT—Overheated			LPT			
		LE Int.	LE Ext.	TE Int.	TE Ext.	LE Int.	LE Ext.	TE	LE Int.	LE Ext.	TE Int.	TE Ext.
γ	Content (%)	residue				residue			residue			
	Size (μm)	84.01		80.48		95.61		93.08	20.62	43.11	35.57	37.81
γ’	Content Share (%)	43.43	49.53	50.36	46.46	51.62	43.07	43.64	41.94		38.73	43.73
	Size (μm)	1.33	3.72	0.22	0.24	1.03	2.24	0.89	1.03	1.07	1.00	1.00
carbides	Content (%)	3.62	3.15	2.37	1.45	9.04	6.32	1.04	2.4		1.56	2.75
	Size (μm)	0.83	1.09	2.42	2.55	0.70	0.75	0.74	1.91	2.52	1.63	2.65

LE—leading edge; TE—trailing edge; Int.—internal area (cooled); Ext.—external area flushed by the exhaust gas stream.

**Table 5:** Strength properties at room temperature of nickel-based superalloys potentially used to construct the HPT blades and LPT blades of the RD33 engine.

Alloy	E [GPa]	HV	R_02_ [MPa]	R_m_ [MPa]	A
Żs-32	244	466	850	880	13.0
Żs-26	253	480	790	1000	6.8
Żs-6U	240	450	770	830	3.0
MAR-M200	230	-	860	960	7.0
Material tested	217	350	678	873	6.9

## Data Availability

Please refer to suggested Data Availability Statements in section “MDPI Research Data Policies” at https://www.mdpi.com/ethics.

## References

[1] Koff B.L. (2004). Gas Turbine Technology Evolution: A Designer’s Perspective. J. Propuls. Power.

[2] Kozakiewicz A. (2009). Comparative analysis of turbojet performance of combat aircraft used in Polish Air Force. Biuletyn WAT.

[3] Igorevich H.I. (2017). Development of economically alloyed high-temperature nickel alloys for single-crystal casting of GTE blades. Ph.D. Thesis.

[4] Trelka M., Bartoszewicz J., Urbaniak R. (2016). Selected problems of RD-33 engine reliability in operation. Combust. Eng..

[5] Carter T.J. (2005). Common failures in gas turbine blades. Eng. Fail. Anal..

[6] Swain B., Mallick P., Patel S., Roshan R., Mohapatra S., Bhuyan S., Priyadarshini M., Behera B., Samal S., Behera A. (2020). Failure analysis and materials development of gas turbine blades. Mater. Today.

[7] Laguna-Camacho J.R., Villagrán-Villegas L.Y., Martínez-García H., Juárez-Morales G., Cruz-Orduña M.I., Vite-Torres M., Ríos-Velasco L., Hernández-Romero I. (2016). A study of the wear damage on gas turbine blades. Eng. Fail. Anal..

[8] Szczeciński S., Balicki W. (2007). Possibilities for Limiting Aircraft Turbine Engine Abrasive Wear of Interior Duct Elements. Trans. Inst. Aviat..

[9] Chamanfar A., Jahazi M., Bonakdar A., Morin E., Firoozrai A. (2015). Cracking in fusion zone and heat affected zone of electron beam welded Inconel-713LC gas turbine blades. Mater. Sci. Eng. A.

[10] Dewangan R., Patel J., Dubey J., Sen P.K., Bohidar S.K. (2015). Gas Turbines Blades—A Critical Review of Failure on First and Second Stages. Int. J. Mech. Eng. Rob. Res..

[11] Bansal L., Rathi V.K., Mudafale K. (2018). A Review on Gas Turbine Blade Failure and Preventive Techniques. Int. J. Eng. Res. Gen. Sci..

[12] Kablov E.N., Opekhov H.G., Tolopaia B.N., Demonis I.M. (2001). Casting heat-resistant alloys and technology of obtaining monocrystalline turbine blades GTE. Light Alloy Technology.

[13] Čerňan J., Janovec M., Hocko M., Cúttová M. (2018). Damages of RD-33 Engine Gas Turbine and their Causes. Transp. Res. Procedia.

[14] Jakubowski R. Teoria Silników Lotniczych—Rozwój i Przegląd Konstrukcji. http://jakubowskirobert.sd.prz.edu.pl/file/MjMsNjcsMjcxLHRlb3JpYV9zaWxuaWtvd19sb3RuaWN6eWNoX3dwcm93YWR6ZW5pZS5wZGY=.

[15] Strotin N.N. (2007). Monitoring and Diagnostics of Technical Condition of Gas Turbine Engines.

[16] Hernas A., Maciejny A. (1989). Żarowytrzymałe Stopy Metali.

[17] Kaibyshev O.A. (1992). Superplasticity of Alloys, Intermetallides and Ceramics.

[18] Kurzydłowski K.J. “Rozwój Materiałów Konstrukcyjnych: Historia Stopów Aluminium” Sesja z Okazji 75-lecia Przeglądu Mechanicznego, 12 luty 2010, Warszawa. www.iinte.edu.pl/PM75/sesja2.pdf.

[19] Siladic M., Rasuo B. On-Condition Maintenance for Non-Modular Jet ENGINES—An Experience, ICAS 2008. Proceedings of the 26th International Congress of The Aeronautical Sciences.

